# Licorice Extract Isoliquiritigenin Increased Cytosol Calcium and Induced Apoptosis in Colon Cancer Cells via Transient Receptor Potential Vanilloid‐1

**DOI:** 10.1002/cam4.70705

**Published:** 2025-02-27

**Authors:** Lin Wang, Qing Li, Lai Kwok Leung, Wing Tak Wong

**Affiliations:** ^1^ School of Life Sciences The Chinese University of Hong Kong Hong Kong China; ^2^ Shenzhen Research Institute The Chinese University of Hong Kong Shenzhen China; ^3^ State Key Laboratory of Agrobiotechnology The Chinese University of Hong Kong Hong Kong China

**Keywords:** calcium, colon cancer, isoliquiritigenin, transient receptor potential vanilloid‐1

## Abstract

**Background:**

Colorectal cancer (CRC) is the third most common malignant tumor, and the fifth cause of cancer‐related death in China, while chemotherapy is the primary strategy for CRC. Transient receptor potential (TRP) channels are non‐selective cation channels while modulating the expression or activity of TRP channels results in the regulation of Ca^2+^ influx. Previous studies have shown that TRP members with altered expression or channel activity are presented in CRC cells, which made them become promising therapeutic targets. Isoliquiritigenin (ISL), one of the major bioactive ingredients from traditional Chinese medicine licorice, was reported to exhibit anti‐cancer properties such as induce apoptosis in CRC cells, but the underlying mechanism was not fully understood, whether its anticancer activity was related to regulating intracellular calcium and TRP channels remains for further investigation.

**Objective:**

The study aims to investigate the effect of ISL on altering cytosol calcium in CRC cells and elucidate its potential molecular mechanism.

**Methods:**

The study was conducted on 2 CRC cell lines HT29 and HCT116. Changes of cytosol calcium was indicated by live cell Ca^2+^ imaging. Expression level of TRPV1 was determined by western blot. Cell apoptosis was detected by flow cytometry with Annexin V‐FITC/PI staining.

**Results:**

ISL significantly increased cytosol calcium in HT29 and HCT116 cells. The ISL‐induced increasing calcium ions were from both calcium influx and intracellular calcium release. ISL co‐culture directly upregulated the expression of transient receptor potential vanilloid‐1 (TRPV1) in colon cancer cells. Inhibition of TRPV1 by capsazepine (CapZ) abrogated the ISL‐induced calcium influx and ISL‐induced apoptosis in HT29 and HCT116 cells.

**Conclusions:**

This study illustrates, for the first time, that ISL increased cytosol calcium concentration and induced apoptosis via TRPV1 in colon cancer cells, giving a new understanding of the underlying mechanism of its anti‐cancer ability and making it a potential regulator for TRPV1.

## Graphical abstract

Isoliquiritigenin (ISL) increased cytosol calcium and induced apoptosis in colon cancer cells via transient receptor potential vanilloid‐1 (TRPV1). CapZ, capsazepine; ISL, isoliquiritigenin; TRPV1, transient receptor potential vanilloid‐1. Created in BioRender.com.

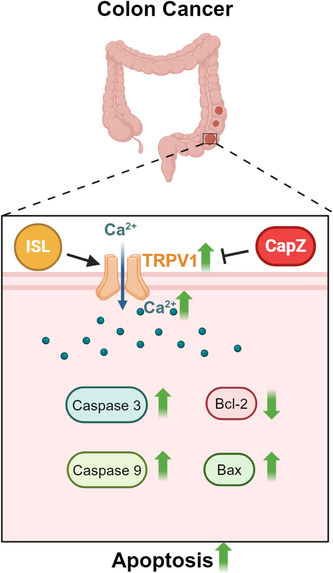

**Abbreviations**
CapZ: capsazepine
CRC: colorectal cancer
DMSO: diemthyl sulfoxide
ER: endoplasmic reticulum
FBS: fetal bovine serum
ISL: isoliquiritigenin
NO: nitric oxide
NPSS: normal physiological saline solution
qPCR: quantitative polymerase chain reaction
TRAIL: tumor necrosis factor related apoptosis‐inducing ligand
TRP: transient receptor potential
TRPV1: transient receptor potential vanilloid‐1

## 1 Introduction

Colorectal cancer (CRC), also known as colon cancer or rectal cancer, is the development of cancer from the colon or rectum, which are parts of the large intestine. According to the statistics, CRC is the third most common malignant tumor globally [1], and the forth cause of cancer‐related death in China [2]. The risk of developing CRC can be influenced by both environmental and genetic factors. Several key risk factors associated with CRC include over the age of 50, overweight or obese, a sedentary lifestyle, smoking, consuming excessive alcohol, and a diet low in fiber yet high in fat [3, 4]. Additionally, the intake of red and processed meats, as well as burnt or charred meats, contributes to increased risk [3].

CRC diagnosed at early stages are highly manageable and frequently respond well to standard treatment methods, leading to potential cures [5]. The primary approach for treatment typically involves surgically removing the affected sections of the colon, which is also applicable to many cases of rectal cancer. The probability of cancer returning is influenced by how deeply the tumor has invaded the bowel wall and whether there is lymph node involvement [5, 6]. Surgical resection proves to be an effective strategy for early‐stage colon cancers, achieving cure rates exceeding 90% for stage I and about 75% for stage II [5]. In patients with stage III cancers, the presence of lymph node involvement raises the chance of recurrence to 60% [7]. Administering a course of chemotherapy based on 5‐Fluorouracil post‐surgery has been shown to reduce recurrence risks to 40% and boost overall survival rates to 60%, establishing this protocol as the standard for stage III patients [7]. However, since existing therapies are still at risk of failure, there is a pressing need for innovative adjuvant treatments to decrease the existing failure rates [5, 7].

In cells, calcium ions (Ca^2+^) can act as second messengers involving in the regulation of gene transcription, cell proliferation, migration and death. Intracellular Ca^2+^ homeostasis is altered in cancer cells and the alteration is involved in tumor initiation, angiogenesis, progression and metastasis [8, 9]. Transient receptor potential (TRP) channels are non‐selective cation channels while modulating the expression or activity of TRP channels results in the regulation of Ca^2+^ influx [10, 11]. Previous studies comparing mRNA expression levels in human CRC tissues versus normal colon mucosa revealed elevated expression of TRPM8, and TRPV6, while the expression of TRPV1, TRPV4, TRPM4, TRPV3, TRPC6, and TRPV5 was lower in CRC tumor tissues compared to normal tissues [12, 13]. Besides, TRPV1 has also been reported to be involved in the process of cancer [14]. Activation of TRPV1 by capsaicin leads to strong calcium increases and induces apoptosis n breast cancer cells [15] and glioma cells [16]. In TRPV1 knockdown mice, the increased carcinogenesis indicating that TRPV1 restricts the initiation and progression of colon cancer [17]. Based on existing results, TRPV1 shows great potential as an innovative therapeutic target for CRC and warrants further investigation.

Licorice, also known as *Glycyrrhiza*, had been served as traditional Chinese medicine for over two thousands years [18]. Mainly extracted from the root of licorice, isoliquiritigenin (2′,4′,4‐trihydroxychalcone, ISL) was considered as one of the most important bioactive ingredients in licorice [19]. Studies had reported that ISL had exhibited anticancer effects in several experimental cancer models [20], including CRC [21, 22], gastric cancer [23], breast cancer [24, 25], lung cancer [26, 27], liver cancer [28] and so on. In CRC, ISL inhibited cell growth via downregulating anti‐apoptotic proteins Bcl‐2 and Bcl‐x(L) [29]. Moreover, ISL had been reported to reduce PGE2 and nitric oxide (NO) production to induce apoptosis in CRC cells [30]. And in the study of Yoshida et al., ISL could help overcome the resistance to tumor necrosis factor (TNF)‐related apoptosis‐inducing ligand (TRAIL), which was also a famous promising anticancer drug [31]. However, the precise mechanisms of its anticancer activity has not been fully investigated. Nevertheless, ISL exhibited its therapeutic properties through regulating intracellular calcium in other disease models such as regulating gastrointestinal motility [32] and exhibited neuroprotective effect against glutamate [33]. But whether ISL could directly alter calcium signaling in CRC cells also remains largely unknown.

This study aims to investigate the potential roles of ISL in altering cytosol calcium concentration in CRC cells and its underlying molecular mechanism.

## 2 Materials and Methods

### 2.1 Drugs and Solutions

Isoliquiritigenin (ISL) was purchased from INDOFINE Chemical Company (Cat No. T‐503, Hillsborough, NJ, USA). ISL was dissolved in Diemthyl Sulfoxide (DMSO, Cat No. 2206‐27‐1, Sigma‐Aldrich Chemical, St. Louis, MO, USA). The TRPV1 inhibitor capsazepine (CapZ) was purchased from Cayman Chemical Co. (Cat No. 10007518, Ann Arbor, MI, USA). Ionomycin was acquired from Cayman Chemical Co. (Cat No. CAY10004974‐10MG, Ann Arbor, Michigan, USA).

### 2.2 Cell Culture

Two CRC cell lines, HT29 and HCT116 were purchased from American Type Culture Collection (ATCC, HT29 Cat No. HTB‐38, HCT116 Cat No. CCL‐247, Manassas, VA, USA). The cells were routinely cultured in McCoy's 5A medium (Cat No. 16600108, Thermo Fisher, Waltham, MA, USA) supplemented with 15% FBS (Cat No. 10270106, Gibco, Grand Island, USA) and 1% Antibiotic‐Antimycotic (Cat No. 15240096, Gibco, Gaithersburg, USA). The cells were placed in a 37°C incubator with 95% O_2_ and 5% CO_2_. Cells were treated with or without 10 μmol/L, 30 μmol/L or 50 μmol/L ISL (dosages of ISL were chose based on previous studies of the induction effects of ISL on apoptosis in CRC cells [22, 31]) with or without 25 μmol/L CapZ in McCoy's 5A medium supplemented with 10% FBS and 1% Antibiotic‐Antimycotic for 24 h. DMSO was used as vehicle control group and maintained at a concentration of not more than 0.1% v/v. There was no exclusion in any of the groups.

### 2.3 Live Cell Ca^2+^ Imaging

CRC cells were cultured in unique confocal dishes. The cells were loaded with 5 μmol/L Fluo‐4 AM (Cat No. F14201, ThermoFisher, Waltham, MA, USA) and 0.02% pluronic acid F‐127 (Cat No. P3000MP, ThermoFisher, Waltham, MA, USA) in normal physiological saline solution (NPSS) containing 1 mmol/L CaCl_2_, 140 mmol/L NaCl, 5 mmol/L KCl, 1 mmol/L MgCl_2_, 10 mmol/L glucose, and 5 mmol/L HEPES and incubated for 20 min at room temperature covered with tin paper. The cells were then washed and bathed in NPSS (with external Ca^2+^) or OPSS (without external Ca^2+^). OPSS solution was prepared with the same formula as NPSS with 0.2 mmol/L EDTA but no Ca^2+^. The cells were illuminated under an excitation wavelength of 488 nm. Changes in cytosolic Ca^2+^ ([Ca^2+^]_i_) fluorescence intensity was monitored in real‐time and recorded using confocal microscope (TCS SP8 MP, Leica, Wetzlar, Germany) [34]. The ratio of [Ca^2+^]_i_ fluorescence normalized to background fluorescence (F1/F0) was calculated. Change in fluorescence was measured as the maximal peak response over the baseline. The solvent DMSO was used as the control, and different doses of ISL was added to induce Ca^2+^ influx. The TRPV1 inhibitor CapZ was co‐cultured with the cells for 24 h before loaded with Fluo‐4AM and F‐127. Ionomycin was a calcium ionophore that acted as a positive control to induce calcium influx and determine the cells were alive.

### 2.4 Western Blot Analysis

Total proteins from CRC cells were extracted with ice‐cold RIPA buffer (Cat. No. P0013B, Beyotime, Shanghai, China) with 1 mmol/L protease inhibitor PMSF (Cat. No. ST506, Beyotime, Shanghai, China) and 10% phosphorylation inhibitor PhosSTOP (Cat. No. 4906837001, Roche Hong Kong Limited, Hong Kong, China). The protein concentration was then determined using the Bio‐Rad Protein Assay Dye Reagent (Cat. No. #5000006, Bio‐Rad Laboratories, Hercules, CA, USA). In total, 30 μg of each perotein samples were electrophoresed through a 10% SDS‐PAGE gel and transferred onto an Immun‐Blot PVDF Membrane (Cat. No. #1620177, Bio‐Rad Laboratories, Hercules, CA, USA). Non‐specific binding sites were blocked with 5% BSA in 0.05% Tween‐20 TBST. The blots were incubated overnight at 4°C with the primary antibody anti‐TRPV1 (1:1000 in 0.05% Tween‐20 TBST, Rabbit, Cat. No. ACC‐030, Alomone, Hadassah Ein Kerem, Jerusalem, Israel) or anti‐β‐actin (1:5000 in 0.05% Tween‐20 TBST, Mouse, Cat. No. sc‐8432, Sigma‐Aldrich, St. Louis, MO, USA), followed by anti‐rabbit IgG secondary antibody HRP conjugate (1:10000 in 0.05% Tween‐20 TBST, Cat. No. A16116, ThermoFisher, Waltham, MA, USA) and anti‐mouse IgG secondary antibody HRP conjugate (1:10000 in 0.05% Tween‐20 TBST, Cat. No. sc‐2359, Santa Cruz Biotechnology, Santa Cruz, CA, USA). Equal protein loading was verified with the use of β‐actin as housekeeping protein. Densitometry was performed with ChemiDoc MP System (BioRad, Hercules, USA). The results were then analyzed with ImageJ (NIH, USA).

### 2.5 Cell Apoptosis Assay

Annexin V‐FITC/PI Apoptosis Detection Kit (Cat. No. F10797, Vazyme, Nanjing, Jiangsu, China) was used to detect the cell apoptosis of CRC cells. The fluorescence of cells was analyzed and measured at 488 nm by a flow cytometer. The excitation wavelength of the flow cytometer is 488 nm; the green fluorescence of Annexin V is detected in the FITC channel; the red fluorescence of PI is detected in the PerCP‐Cy5.5 channel and 20,000 events are collected for each sample. FlowJo Software (v10.8, BD Life Sciences, Franklin Lakes, NJ, USA) was used for data analysis. FITC is the abscissa and PerCP‐Cy5.5 is the ordinate. According to FITC and PerCP‐Cy5.5 fluorescence values, the cut‐off between positive and negative cells can be given to set the gate. Cells were divided into four subpopulations: living cells were double negative (Annexin V^−^/PI^−^); early apoptotic cells were Annexin V‐FITC single positive (Annexin V^+^/PI^−^); late apoptotic cells were double positive for Annexin V‐FITC and PI (Annexin V^+^/PI^+^); dead cells that have been mechanically damaged were PI single positive (Annexin V^−^/PI^+^).

### 2.6 Quantitative Polymerase Chain Reaction (qPCR) Analysis

Total RNA was extracted from HCT116 and HT29 cells with RNAiso plus reagents (Cat No. 9109, Takara, Shiga, Japan). The Spectrophotometer (NanoDrop One, Thermo fisher Scientific) was used to determine RNA concentration. Afterwards, 1000 ng RNA was reversed into cDNA using PrimeScript MasterMix kit (Cat No. RR036B, Takara, Shiga, Japan). Quantitative polymerase chain reaction (qPCR) experiments were then performed using the qPCR system (BioRad, CFX96, Hercules, USA) with TB Green Premix Ex Taq kit (Cat No. RR420A, Takar, Japan) according to the manufacturer's protocol. mRNA expression levels were normalized to the housekeeping gene β‐actin. The Delta–delta CT method was used to analyze the results.

### 2.7 Statistical Analysis

Results were presented as mean ± standard error of the mean (SEM). The n value was three or five independent experiments. Comparisons among different groups were performed using one‐way ANOVA followed by Tukey's test. Data were analyzed using GraphPad Prism software (Version 9.0, San Diego, CA, USA). *p* < 0.05 was regarded as statistically significant.

## 3 Results

### 3.1 Isoliquiritigenin Dose‐Dependently Increased Intracellular Calcium Concentration in Colon Cancer (CRC) Cell

**FIGURE 1:**
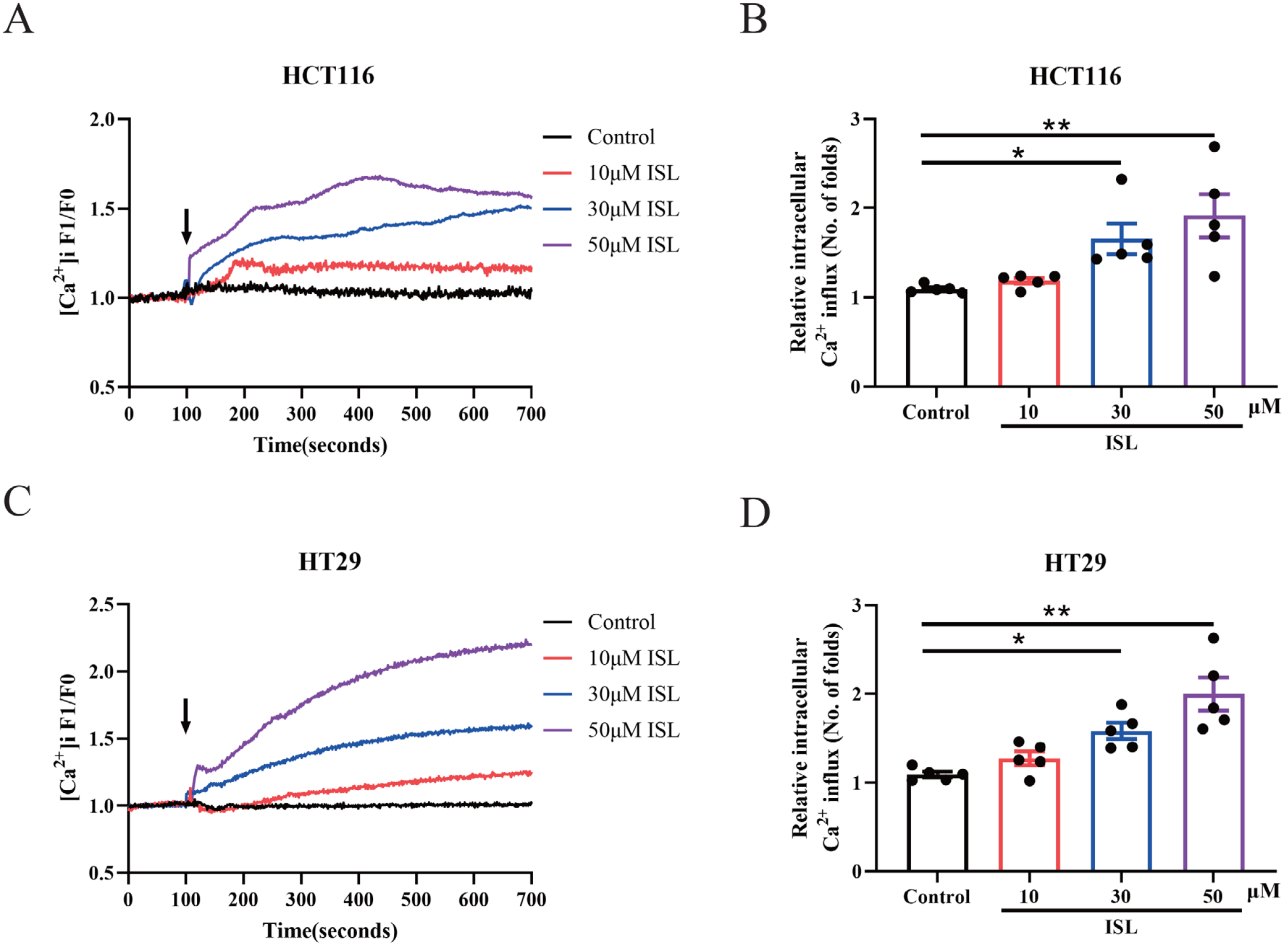
ISL dose‐dependently increases intracellular calcium concentration in colon cancer cells. HT29 cells and HCT116 were seeded in confocal dish and Fluo‐4 AM was used to label cytosolic Ca^2+^ and monitored intracellular calcium real‐time. (A, B) HCT116 cells. (C, D) HT29 cells. (A, C) Representative curves of changes of intracellular calcium in real time. (B, D) The peak levels of intracellular calcium levels. F1 denotes [Ca^2+^]i fluorescence during the whole period of recording while F0 denotes [Ca^2+^]i fluorescence at baseline (before drug addition). Data represent means ± SEM of five experiments. *p* values were determined using One‐way ANOVA. *p < 0.05, **p < 0.01.

Whether ISL increases intracellular calcium was first examined by live cell calcium imaging. The two CRC cell lines HCT116 and HT29 cells were loaded with extra Fluo‐4 AM labeled Ca^2+^ buffer and monitored intracellular calcium concentrations in real‐time. ISL was directly added to the cells under the microscope in real‐time. The results showed that ISL dose‐dependently increases intracellular calcium concentrations in both HCT116 (Figure 1A,B) and HT29 cells (Figure 1C,D). These findings collectively illustrate that ISL increases intracellular calcium concentrations in CRC cells.

### 3.2 The ISL‐Induced Enhanced Calcium Signal Was From Both Increasing Calcium Influx and Intracellular Calcium Release

**FIGURE 2:**
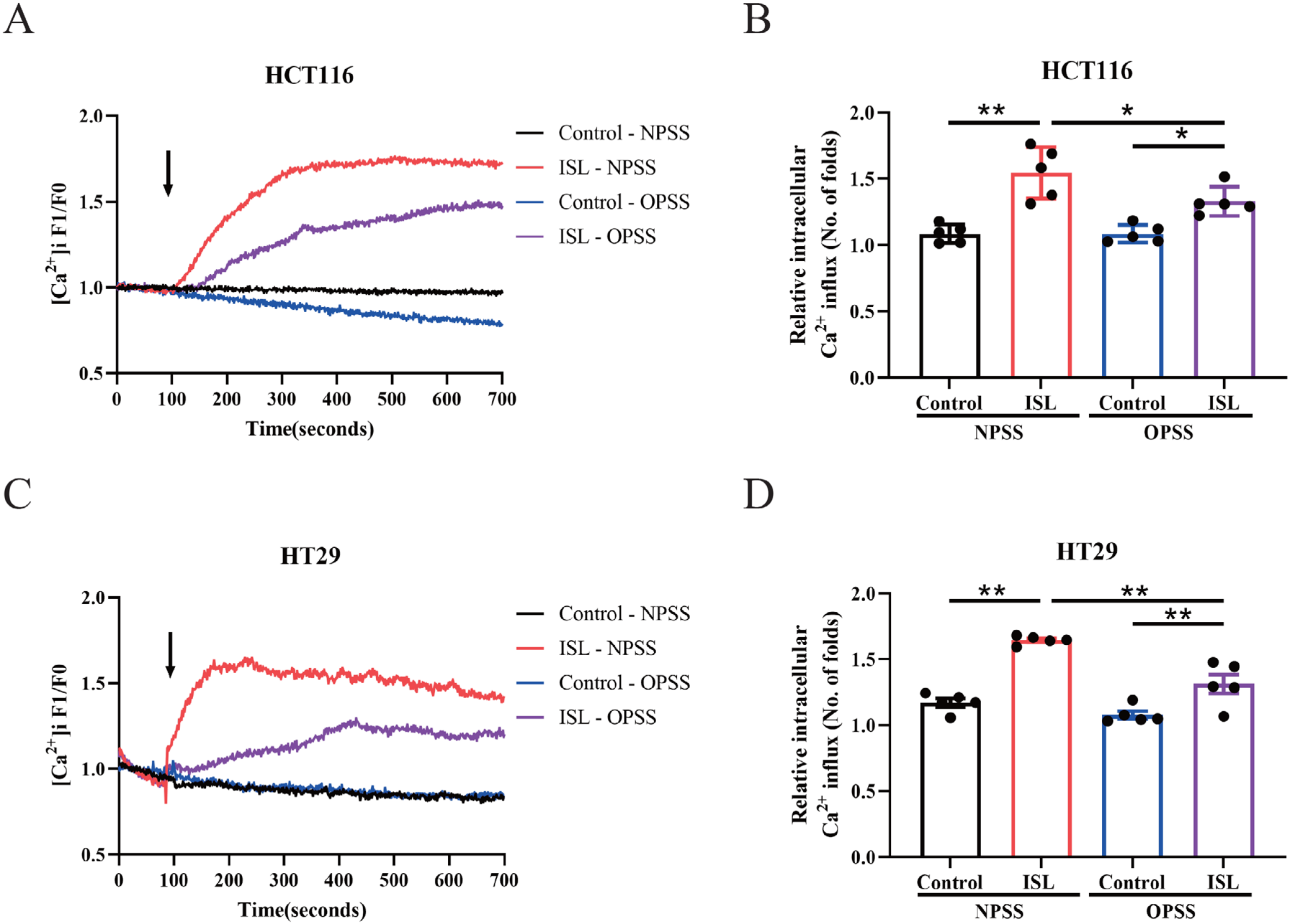
The ISL‐induced enhanced calcium signal is from both increasing calcium influx and intracellular calcium release. HT29 cells and HCT116 were seeded in confocal dish and Fluo‐4 AM was used to label cytosolic Ca^2+^ and monitored intracellular calcium real‐time. (A, B) HCT116 cells. (C, D) HT29 cells. (A, C) Representative curves of changes of intracellular calcium in real time. (B, D) The peak levels of intracellular calcium levels. F1 denotes [Ca^2+^]i fluorescence during the whole period of recording while F0 denotes [Ca^2+^]i fluorescence at baseline (before drug addition). Treatments were listed as follows: ISL, 30 μmol/L; NPSS, normal medium; OPSS, calcium‐free medium. Data represent means ± SEM of five experiments. *p* values were determined using one‐way ANOVA. *p < 0.05, **p < 0.01.

Since the increasing cytosol calcium comes from both calcium influx from extracellular pool and intracellular calcium release from endoplasmic reticulum (ER), we then evaluated where were the increasing calcium from by incubating the CRC cells with calcium free medium (OPSS). In both normal medium (NPSS) and calcium free medium groups, ISL significantly increases intracellular calcium concentrations in CRC cells (Figure 2). However, there's also a significant difference between the normal medium group and calcium free medium group in HCT116 (Figure 2A,B) and HT29 (Figure 2C,D) cells after loading ISL, showing that intracellular calcium release was not the only resource of ISL‐induced enhanced calcium signal. Taken together, the above findings indicated that the ISL‐induced enhanced calcium signal is from both increasing calcium influx and intracellular calcium release in CRC cells.

### 3.3 ISL Increases the Expression Level of TRPV1 in CRC Cell

**FIGURE 3:**
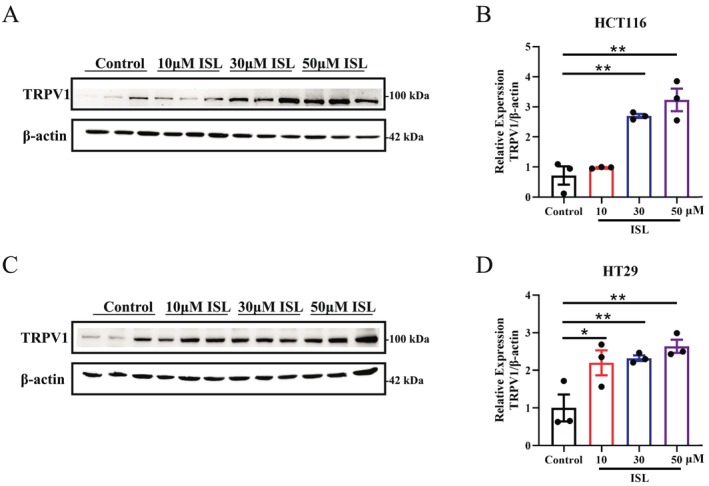
ISL increases TRPV1 expression level in colon cancer cells. western Blot (WB) was used to detect the expression of TRPV1. (A) Representative WB image in HCT116 cells. (B) Statistical graph of WB results in HCT116 cells. (C) Representative WB image in HT29 cells. (D) Statistical graph of WB results in HT29 cells. Data represent means ± SEM of three experiments. *p* values were determined using One‐way ANOVA. *p < 0.05, **p < 0.01.

As the most famous TRPV1 activator, capsaicin had been reported to have the ability to active TRPV1 activity [35] as well as upregulate TRPV1 expression level [36]. Based on that, we then examined the expression level of TRPV1 by western blot to investigate whether ISL has similar biological effects as capsaicin. The results indicated that ISL treatment dose‐dependently increases the protein expression level of TRPV1 in HCT116 (Figure 3A,B) and HT29 cells (Figure 3C,D), which showed a similar trend as calcium imaging results in the two CRC cell lines. To conclude, the above findings demonstrate that ISL increases the expression level of TRPV1 in CRC cells, indicating that ISL has the possibility of similar biological effects to capsaicin.

### 3.4 Inhibition of TRPV1 by Capsazepine Abrogates the ISL‐Induced Calcium Influx in CRC Cell

**FIGURE 4:**
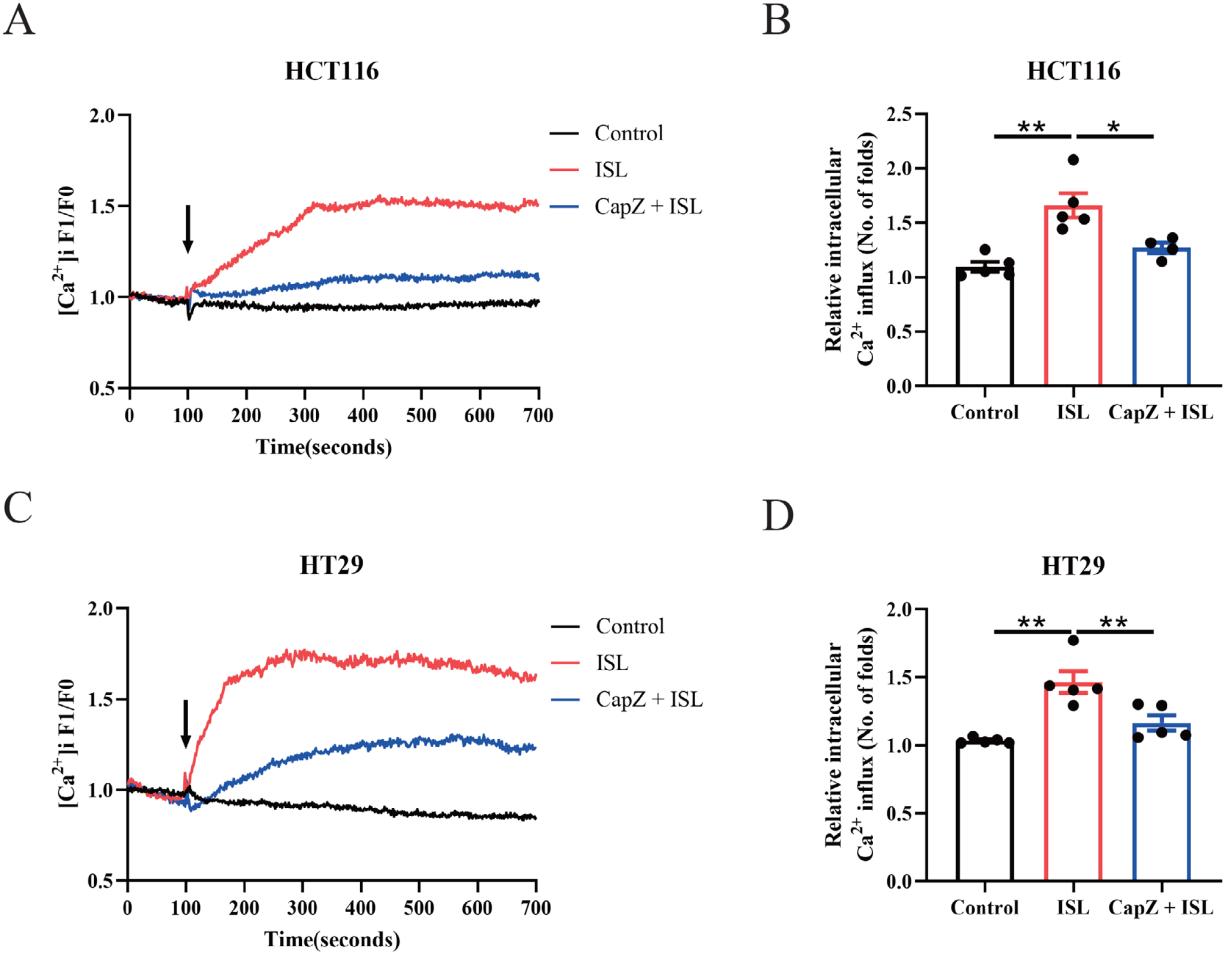
Inhibition of TRPV1 abrogates the ISL‐induced calcium influx. HT29 cells and HCT116 were seeded in confocal dish and Fluo‐4 AM was used to label cytosolic Ca^2+^ and monitored intracellular calcium real‐time. (A, B) HCT116 cells. (C, D) HT29 cells. (A, C) Representative curves of changes of intracellular calcium in real time. (B, D) The peak levels of intracellular calcium levels. F1 denotes [Ca^2+^]i fluorescence during the whole period of recording while F0 denotes [Ca^2+^]i fluorescence at baseline (before drug addition). Treatments were listed as follows: ISL—30 μmol/L; CapZ (TRPV1 blocker)—25 μmol/L. Data represent means ± SEM of five experiments. *p* values were determined using one‐way ANOVA. *p < 0.05, **p < 0.01.

The effects of ISL on TRPV1 was further investigated by conducting live cell Ca^2+^ imaging with co‐incubation of TRPV1 specific inhibitor CapZ. Our results shows that in CapZ co‐culture groups, ISL fails to induce increasing calcium influx in HCT116 (Figure 4A,B) and HT29 cells (Figure 4C,D). In conclusion, the results indicates that inhibition of TRPV1 by CapZ abrogates the ISL‐induced calcium influx, illustrating that ISL induces increasing calcium influx through TRPV1.

### 3.5 Inhibition of TRPV1 Eliminates the Apoptosis Induced by ISL in CRC Cell

**FIGURE 5:**
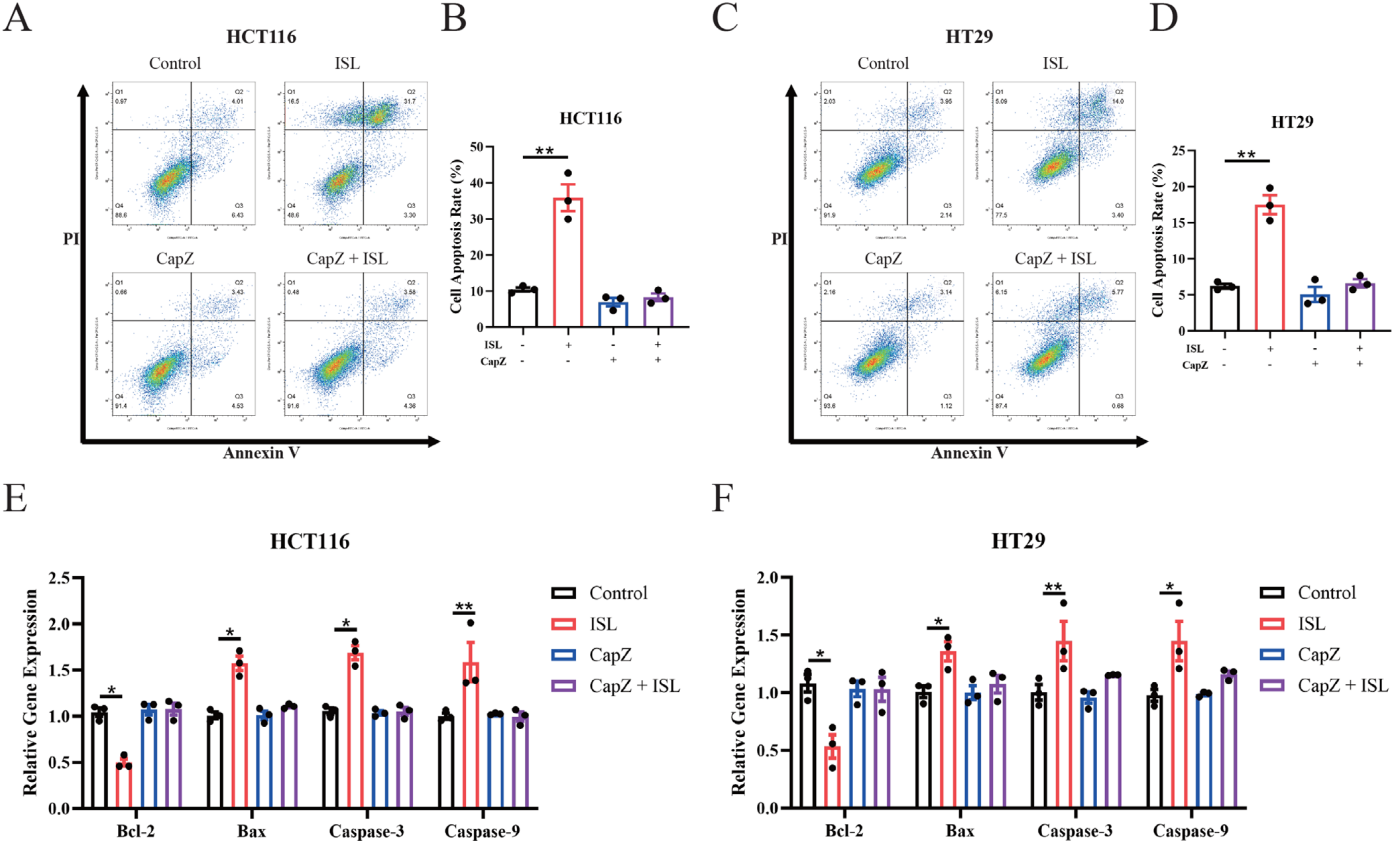
Inhibition of TRPV1 abrogates the apoptosis‐inducing effects of ISL. Cells were treated with or without 30 μmol/L ISL and 25 μmol/L CapZ for 24 h. (A–D) Flow cytometry analysis with Annexin V‐PI staining was performed to evaluate the percentage of apoptotic cells of (A) HCT116 cells and (C) HT29 cells. (B) Analysis on cell apoptosis results of HCT116 cells. (D) Analysis on cell apoptosis results of HT29 cells. Q1: Annexin V^−^/PI^+^, necrotic cells and mechanically damaged cells. Q2: Annexin V^+^/PI^+^, late apoptotic cells. Q3: Annexin V^+^/PI^−^, early apoptotic cells. Q4: Annexin V^−^/PI^−^, living cells. The cell apoptosis rate was the proportion of Q2 and Q3 cell population to total cell number. (E, F) qPCR analysis demonstrates that the downregulation of Bcl‐2 by ISL treatment in HCT116 cells (E) and HT29 cells (F) is abrogated by CapZ. The upregulation of Bax, Caspase 3 and Caspase 9 by ISL treatment in HCT116 cells (E) and HT29 cells (F) are also abolished by CapZ. mRNA expressions were normalized with β‐actin. Data represent means ± SEM of three experiments. *p* values were determined using one‐way ANOVA. *p < 0.05, **p < 0.01.

Previous studies indicated that ISL exhibits anti‐cancer properties [20] through inhibiting proliferation [37], invasion [21] and migration [21] and inducing apoptosis [31] and autophagy [22] in CRC cells, but the underlying mechanisms were not fully understood [20]. Besides, TRPV1 has also been reported to be involved in the process of cancer [14]. Activation of TRPV1 by capsaicin leads to strong calcium increases and induces apoptosis in breast cancer cells [15] and glioma cells [16]. In TRPV1 knockdown mice, the increased carcinogenesis indicating that TRPV1 restricts the initiation and progression of CRC [17], but whether TRPV1 restricts CRC development by inducing apoptosis remains unclear. Base on these, we then investigated whether ISL induces apoptosis through TRPV1 in CRC cells by conducting flow cytometry with CapZ co‐incubation. Our results showed that rate of apoptotic cells were increased after 24 h ISL treatment in both HCT116 (Figure 5A,B) and HT29 cells (Figure 5C,D). Conversely, co‐culture with CapZ abrogates ISL‐induced apoptosis in HCT116 (Figure 5A,B) and HT29 cells (Figure 5C,D). Furthermore, since apoptosis occurs through a series of steps involving the cleavage and activation of caspases in a specific order, while ISL has been reported to induce apoptosis in CRC cells via activating Caspase 3 and Caspase 9 [31]. Additionally, in CRC cells [38] and ovarian cancer cells [39, 40], ISL induced apoptosis through upregulating anti‐apoptotic regulator Bax (also known as Bcl‐2‐like protein 4) and downregulating pro‐apoptotic regulator Bcl‐2. Therefore, we also measured the mRNA levels of Bcl‐2, Bax, Caspase 3 and Caspase 9 after treatment with ISL and CapZ using qPCR. Encouragingly, our results revealed that ISL treatment significantly downregulated the mRNA level of Bcl‐2 and upregulated the mRNA levels of Bax, Caspase 3 and Caspase 9 in both HCT116 cells (Figure 5E) and HT29 cells (Figure 5F), while CapZ treatment abolished these effects of ISL. Overall, these above findings collectively demonstrate that ISL induces apoptosis via TRPV1 in CRC cells.

## 4 Discussion

Our study demonstrates that ISL dose‐dependently increased the cytosol calcium concentration, while the increasing cytosol calcium ions were from both extracellular calcium influx and intracellular calcium release. Mechanistically, ISL significantly increased the expression level of TRPV1, while inhibition of TRPV1 abrogated the ISL‐induced calcium influx as well as ISL‐induced apoptosis, indicating that ISL increases intracellular calcium ions and apoptosis via upregulating TRPV1.

Several studies had demonstrated that tumorigenic pathways had been proved to be associated with altered expression level or abnormal activation of Ca^2+^ channels, transporters or Ca^2+^‐ATPases [8]. Our study firstly reported that ISL increases cytosol calcium in CRC cells, giving new understanding to investigate the underlying mechanism of the anticancer properties of ISL. Despite other Ca^2+^ channels, TRPV1 had also been reported to restrict the initiation and progression [17] or inducing apoptosis [41] in CRC. To the best of our knowledge, the study is the first to discover ISL alters cytosol calcium and induces apoptosis via regulating TRPV1. The underlying mechanism that ISL induces apoptosis in CRC cellsvia activating Caspase 3 and Caspase 9 [31] or through upregulating anti‐apoptotic regulator Bax and downregulating pro‐apoptotic regulator Bcl‐2 [38] have also been verified and our finding firstly demonstrated that ISL downregulates Bcl‐2 as well as upregulates Bax, Caspase 3 and Caspase 9 through TRPV1. Since ISL has also been reported to induce apoptosis in CRC through reducing PGE2 and NO production [30] or through inhibition of p62/SQSTM1 [22], whether these potential mechanisms are also related to intracellular calcium signaling and TRPV1 as proposed in this study also needs further investigation. Besides, apoptosis is not the only type of cell death, while necroptosis, autophagy, and pyroptosis are also possible ways of cell death [42] induced by ISL. Due to the limitation of the Annexin V‐FITC/PI staining method used in flow cytometry in this study, which can only detect apoptosis, whether ISL also promotes other three forms of cell death in CRC cells through calcium signaling and TRPV1 requires further investigation.

This study represents the first investigation to evaluate the effects of ISL on cytosol calcium and TRPV1 in CRC cell. However, the downstream molecular mechanism by which ISL induces apoptosis through TRPV1 remains further investigated. Furthermore, this study did not examine all the cancer‐related biological properties of ISL such as proliferation, progression and migration. Therefore, further investigations can be conducted to characterize whether ISL treatment exhibits other anticancer properties including inhibiting proliferation, progression and migration through TRPV1. These elucidations will provide novel in‐sights into the therapeutic effects of ISL on CRC.

## 5 Conclusion

In conclusion, our results demonstrate that ISL directly increases intracellular calcium concentration in CRC cells via increasing calcium influx as well as increasing intracellular calcium release. Moreover, ISL significantly increases the expression level of TRPV1, while inhibition of TRPV1 by CapZ abrogates the effects of ISL on increasing calcium influx, indicating that ISL may alter intracellular calcium via TRPV1. Additionally, inhibition of TRPV1 by CapZ also abrogates the effect of ISL on inducing apoptosis in CRC cells. These findings highlight the potential of ISL as a promising TRPV1 activator in endothelial cells, providing a new insight for further investigation of the therapeutic potentials of ISL and TRPV1.

## Author Contributions

**Lin Wang:** writing – original draft, writing – review and editing, methodology, investigation, data curation, visualization, validation, formal analysis. **Qing Li:** methodology, investigation. **Lai Kwok Leung:** conceptualization, formal analysis, methodology, resources, supervision, validation, visualization. **Wing Tak Wong:** conceptualization, formal analysis, funding acquisition, methodology, project administration, resources, supervision, validation, visualization, writing – original draft, writing – review and editing.

## Conflicts of Interest

The authors declare no conflicts of interest.

## Data Availability

The data that support the findings of this study are available from the corresponding author upon reasonable request.
